# To eradicate or not? *Helicobacter pylori* in patients with inflammatory bowel disease: an updated systematic review and meta-analysis

**DOI:** 10.3389/fmed.2026.1757356

**Published:** 2026-02-03

**Authors:** Yuzhen Bi, Limin Zhou, Shunhai Zhou, Yan Sun, Jun Zhang

**Affiliations:** 1 Department of Gastroenterology, Chun’an County First People’s Hospital (Zhejiang Provincial People’s Hospital, Chun’an branch), Hangzhou, Zhejiang, China; 2 Second School of Clinical Medicine, Zhejiang Chinese Medical University, Hangzhou, Zhejiang, China; 3 Key Laboratory of Gastroenterology of Zhejiang Province, Zhejiang Provincial People’s Hospital (Affiliated People's Hospital, Hangzhou Medical College), Hangzhou, Zhejiang, China; 4 Department of Gastroenterology, First Affiliated Hospital of Zhejiang Chinese Medical University (Zhejiang Provincial Hospital of Chinese Medicine), Hangzhou, Zhejiang, China

**Keywords:** Crohn disease, *Helicobacter pylori*, inflammatory bowel diseases, negative association, ulcerative colitis

## Abstract

**Background and aims:**

The management of *Helicobacter pylori* (*H. pylori*) in patients with inflammatory bowel disease (IBD) presents a common clinical dilemma. While standard guidelines recommend *H. pylori* eradication to prevent gastric pathology, emerging evidence suggests a potential complex relationship with IBD. This study aims to critically evaluate this relationship through an updated systematic review and meta-analysis to inform clinical decision-making.

**Methods:**

A comprehensive literature search on four major databases, PubMed, Embase, Medline, and Web of Science, was conducted, and all records before July 10th, 2025, were retrieved for screening. Pooled odds ratios (OR) with 95% confidence intervals (CIs) were calculated using STATA 18 software and a random-effects model (Restricted Maximum Likelihood, REML). Subgroup analyses, meta-regression, heterogeneity, sensitivity, and publication bias analyses were performed.

**Results:**

Analysis of 44 studies involving 14,100 IBD patients and 291,352 controls revealed a significantly lower prevalence of *H. pylori* infection in IBD patients compared to controls (13.48% vs. 10.87%; OR: 0.43, 95% CI: 0.35–0.53, *p* < 0.01). This negative association was particularly strong for Crohn’s disease (OR: 0.36, 95%CI: 0.28–0.45) and in Eastern populations (OR: 0.34, 95%CI: 0.26–0.40). Heterogeneity was high (I^2^ = 84.93%), but sensitivity analysis confirmed the robustness of the findings. No significant publication bias was detected.

**Conclusion:**

This meta-analysis demonstrates a significant negative association between *H. pylori* infection and IBD, particularly in patients with Crohn’s disease and those of Eastern population. Furthermore, *H. pylori* may exert a potential immunomodulatory role in IBD.

**Systematic review registration:**

https://www.crd.york.ac.uk/PROSPERO/view/CRD42024567688, CRD42024567688.

## 1 Introduction

The management of *Helicobacter pylori* infection in patients with inflammatory bowel disease (IBD) represents a growing clinical challenge. IBD is a chronic inflammatory condition of the gastrointestinal tract that requires long-term management (1, 2). The highest incidence rate of the disease occurs in early adulthood (3). The three major forms of IBD are Crohn’s disease (CD), Ulcerative colitis (UC), and IBD unclassified (IBDU). Currently, the definitive etiology of IBD is still unknown. However, genetic susceptibility of the host, intestinal microbiota, other environmental factors (e.g., diet, smoking, and physiological stress), and immunological abnormalities are aspects generally associated with the onset of IBD. Common controlling medications to stop the disease from developing into active phase, including 5-aminosalicylates, steroids, Immunosuppressive drugs, and biological agents (monoclonal antibodies, etc.), only target the inflammatory process and are often unsatisfactory in their results.

*H. pylori* infection can lead to upper gastrointestinal disorders, including chronic gastritis, peptic ulcer disease, gastric mucosa-associated lymphoid tissue (MALT) lymphoma, and gastric cancer (4). The Kyoto global consensus on *H. pylori* gastritis advocates eradication therapy for all infected individuals unless there are competing considerations, despite potential adverse effects including obesity, allergy, and perturbation of the intestinal microbiota (5).

The potential protective effect of *H. pylori* for IBD has been suspected after a series of observational studies spanning nearly three decades. Ormand et al. were the first to examine the relationship between *H. pylori* infection and different forms of gastritis, including Crohn’s Disease, in 1991 (6). In 1994, El-Omar et al. explicitly revealed a lower prevalence of *H. pylori* among IBD patients (7). The most recently published study related to the topic was by Garka-Pakulska et al., who took it a step further by comparing the endoscopic presentation between *H. pylori* positive and negative individuals with IBD (8).

The abundance of original studies has provided a valuable opportunity for statistical analysis. Several systematic reviews and meta-analyses exploring a possible connection between *H. pylori* and IBD have been published (9–19), with one in 2023 analyzing the situation in the child population. Most of these favored a potential protective role of *H. pylori*, except for the aforementioned pediatric study, which reported no significant correlation between H pylori infection and IBD (11). The objective of this systematic review and meta-analysis is to address this clinical question by providing an updated, comprehensive assessment of the relationship between *H. pylori* infection and IBD, incorporating the most recent evidence to guide clinical decision-making.

## 2 Methods

This systematic review and meta-analysis was conducted and reported under the requirements of the 2020 Preferred Reporting Items for Systematic Review and Meta-analysis (PRISMA) statement (20). The protocol of this study was registered in the International Prospective Register of Systematic Reviews, PROSPERO, registration ID CRD42024567688.

### 2.1 Search strategy

Literature search was performed in four major medical-related databases: PubMed, Embase, Medline, and Web of Science, with language restricted to English and all records dated before July 10th, 2025, retrieved for screening. The search syntax was constructed based on Medical Subject Headings (MeSH) and relevant free words, including but not limited to ‘Crohn Disease’, ‘Crohn’s disease’, ‘Crohn’s Enteritis’, ‘Regional Enteritis’, ‘Granulomatous Enteritis’, ‘Terminal Ileitis’, ‘Colitis, Ulcerative’, ‘Idiopathic Proctocolitis’, ‘Ulcerative Colitis’, ‘Colitis Gravis’, ‘Inflammatory Bowel Diseases’, ‘*Helicobacter pylori*’, ‘*Helicobacter nemestrinae*’, and ‘*Campylobacter pylori*’, connected by Boolean operators. Supplementary material S1 provides the complete search syntax used for all databases.

### 2.2 Study selection

Observational studies regarding the association between *H. pylori* infection and IBD (CD, UC, or IBDU) were selected meticulously by two reviewers (Y. S. and Y. B.) who were trained on the eligibility criteria using EndNote X9 (Clarivate, London). The “find duplicates” function in EndNote X9 was utilized before the two reviewers independently reinspected the whole list of entries manually for any duplicates. Independent screening of the articles based on the relevance of the title, abstract, and full text was performed by the two reviewers, with any disagreements in the opinion were settled by consulting another senior reviewer (J. Z.) and consensus.

The inclusion criteria for studies were as follows: (1)The study was an observational study of a cohort, case–control, or cross-sectional design, carried out not on an entirely pediatric population; (2) The study included patients with the diagnosis of IBD, including CD, UC, and IBDU; (3) The status of *H. pylori* infection of study subjects were detected using one of the following techniques: urease breath test(UBT), rapid urease test(RUT), polymerase chain reaction(PCR), stool antigen testing, serological examination with enzyme-linked immunosorbent assay(ELISA), histology, or culture; (4) *H. pylori* infection status was reported in either numbers of infected individuals or OR with 95% CI; (5) The study was published as a peer-reviewed full text article. Exclusion criteria comprised study that: (1) performed on animal models or cell strains; (2) included primarily a pediatric population; (3) without the availability of the complete data; (4) not in the English language; (5) of publication types of conference proceedings, comments, letters, editorials.

### 2.3 Data extraction

Reviewers (Y. S. and Y. B.) independently extracted the data using a pre-designed Microsoft Excel sheet. Disagreements were settled by consulting another senior reviewer (J. Z.) and consensus. The first author’s family name, journal title, article title, country, region, the time of publication for each study, the definition of IBD adopted in the study, the categories of IBD, the detection methods for *H. pylori*, the sample size of each study, the age of participants, and study outcome (numbers, or ORs with 95% CI) were extracted.

### 2.4 Risk of bias assessment

Risk of bias assessment was performed by reviewers (Y. S. and Y. B.) using the Newcastle-Ottawa-Scale (NOS), a judgment framework based on the selection of the study groups, and the measurement of the exposure status, with a maximum of 9 stars. A study that scored 7 stars or higher was defined as high quality. Any disagreements in the assessment result were settled by consulting the senior reviewer (J. Z.) and consensus.

### 2.5 Data analysis

Data were analyzed using STATA 18 (StataCorp LP, College Station, Texas). Odds ratio (OR) with a 95% confidence interval (CI) was adopted as the effect measure for all meta-analyses. Heterogeneity was measured with the Cochrane Q *p*-value and the Higgins I^2^ statistics (21). Significant heterogeneity was defined as a Cochrane Q p-value < 0.05 or Higgins I^2^ > 50%. Considering the original studies varied greatly in various aspects, including the demographical composition of subjects, subtypes and definition of IBD, and *H. pylori* testing methods, a random-effect model was used as a default for data synthesis. Statistical significance was defined as a *p*-value less than 0.05. Sensitivity analyses were conducted by excluding low-quality studies and those with a sample size of less than 100, and a leave-one-out sensitivity analysis was additionally performed to evaluate the robustness of the pooled results. Meta-analysis and leave-one-out sensitivity analysis results were visually presented with forest plots. Data were stratified according to: (a) IBD subtype; (b) Study design; (c) Age stratification; (d) Age difference; (e) Ethnicity; (f) *H. pylori* detection method; (g) Definition of IBD; (h) Quality based on the risk of bias; and (i) Source of control, to enable subgroup analyses and meta-regression to determine the underlying source of heterogeneity. Difference between subgroups was analyzed with the test of group differences in STATA. Publication bias was assessed visually with a funnel plot and quantitatively with Egger’s test.

## 3 Results

### 3.1 Study selection

**Figure 1:**
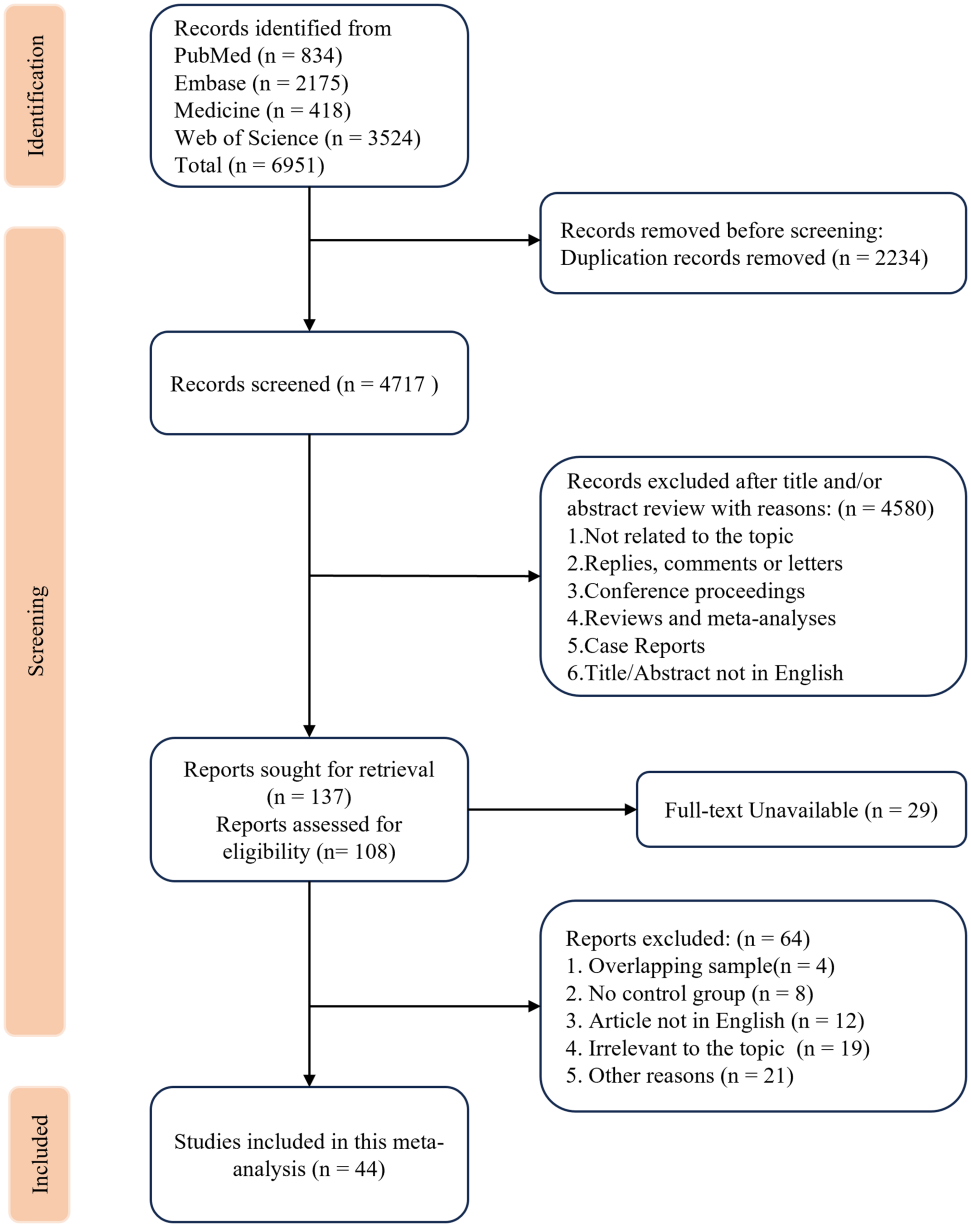
PRISMA flow diagram of the current systematic review and meta-analysis (Comprehensive search of the databases was completed on July 10th, 2025).

Database search yielded 6,951 entries (Figure 1). After removing 2,234 duplicates, 4,717 entries with unique titles were screened. 137 studies investigating the relation between *H. pylori* infection and IBD were retrieved for their potential eligibility. Of these, 46 studies met the inclusion criteria. After carefully reviewing the original studies, two studies conducted by Sonnenberg et al. were found to have potentially overlapping sample populations with another two and were therefore excluded (22, 23). Thus, 44 studies with complete full-text articles and available data were finally included.

### 3.2 Characteristics of the included studies

The 44 included studies all had a case–control design. Total case and control numbers were 14,100 and 291,352, respectively. Among these, 36 studies included CD (7, 8, 24–56), 29 included UC (7, 26–29, 31–33, 37, 38, 40–42, 45, 47–50, 53–63), and 5 included IBDU (40, 50, 54, 56, 64). Due to the scarcity of original studies analyzing IBDU as an individual entity, statistics of studies conducted on Microscopic Colitis (MC) have been included and merged to the IBD unclassified group due to its unique characteristics, which share some similarities with IBDU. Two studies conducted by Sonnenberg et al. had overlapping sample populations, and was therefore excluded (22, 23). Two studies did not differentiate between the subtypes of IBD (65, 66). Two studies included by Oliveira et al. shared the same control group of 76 individuals (44, 60). The majority of studies (24/44) were conducted on the Western population, with 12 on the Eastern population, 8 on the other population. IBD was defined using a reliable medical registry in 26 studies and colonoscopy/biopsy in 14. Four studies did not detail the definition of IBD in the respective settings. Regarding the ascertainment of *H. pylori* infection, 15 of the included studies incorporated UBT into the testing scheme, 2 adopted stool antigen testing, 17 included invasive testing methods that required endoscopic biopsy, and 16 utilized serology. Nine studies used more than one detection methods for *H. pylori*. The general information of the included studies is listed in Table 1.

**Table 1:** Main characteristics of the included studies in this meta-analysis on *Helicobacter pylori* infection and IBD.

Author	Publishing year	Journal	Region	NOS Score	IBD diagnosis	Type	Hp diagnosis method	Case	Control	Total
El-Omar et al. (7)	1994	Gut	UK	6	Not Mentioned	CD/UC	Serology/14C-UBT/histology	110	100	210
Halme et al. (33)	1996	Journal of Clinical Pathology	Finland	7	Registry/Medical Record	CD/UC	Serology	200	100	300
Meining et al. (34)	1997	Scandinavian Journal of Gastroenterology	Germany	6	Colonoscopy & Biopsy	CD	Histology	36	36	72
Oberhuber et al. (35)	1997	Gastroenterology	Germany	6	Registry/Medical Record	CD	Histology	75	193	268
Parente et al. (61)	1997	Scandinavian Journal of Gastroenterology	Italy	7	Registry/Medical Record	CD/UC	Serology	216	216	432
M. J. Wagtmans et al. (36)	1997	Scandinavian Journal of Gastroenterology	Netherlands	7	Registry/Medical Record	CD	Serology	386	277	663
D’Incà et al. (26)	1998	Digestive Diseases and Sciences	Italy	6	Registry/Medical Record	CD/UC	Histology	108	43	151
Duggan et al. (29)	1998	Gut	UK	7	Registry/Medical Record	CD/UC	Serology	257	174	431
Parente et al. (37)	2000	American Journal of Gastroenterology	Italy	7	Registry/Medical Record	CD/UC	13C-UBT/histology	220	141	361
Pearce et al. (38)	2000	European Journal of Gastroenterology & Hepatology	UK	5	Registry/Medical Record	CD/UC	Serology/13C-UBT	93	40	133
Matsumura et al. (39)	2001	Journal of Gastroenterology	Japan	5	Registry/Medical Record	CD	Serology	90	525	615
Väre et al. (40)	2001	Scandinavian Journal of Gastroenterology	Finland	7	Registry/Medical Record	CD/UC/IBDU	Serology	296	70	366
Feeney et al. (31)	2002	European Journal of Gastroenterology and Hepatology	UK	6	Registry/Medical Record	CD/UC	Serology	276	276	552
Piodi et al. (41)	2003	Journal of Clinical Gastroenterology	Italy	7	Registry/Medical Record	CD/UC	13C-UBT	72	72	144
Prónai et al. (42)	2004	Helicobacter	Hungary	6	Registry/Medical Record	CD/UC	13C-UBT	133	200	333
Oliveira et al. (60)	2004	Journal of clinical microbiology	Brazil	5	Colonoscopy & Biopsy	UC	Serology/13C-UBT	42	74	116
Moriyama et al. (43)	2005	Alimentary Pharmacology & Therapeutics	Japan	7	Registry/Medical Record	CD	13C-UBT	29	7	36
Oliveira et al. (44)	2006	Helicobacter	Brazil	6	Colonoscopy & Biopsy	CD	Serology/13C-UBT	43	74	117
Ando et al. (24)	2008	Journal of Gastroenterology and Hepatology	Japan	7	Not Mentioned	CD	13C-UBT/Histology	38	12	50
Ando et al. (45)	2008	Digestion	Japan	6	Not Mentioned	UC/CD	13C-UBT	52	26	78
Laharie et al. (46)	2009	Alimentary Pharmacology & Therapeutics	France	6	Colonoscopy & Biopsy	CD	PCR	73	92	165
Lidar et al. (47)	2009	Contemporary Challenges in Autoimmunity	Italy	8	Not Mentioned	IBD	Serology	119	98	217
Song et al. (48)	2009	The Korean journal of gastroenterology	Korea	7	Registry/Medical Record	CD/UC	UBT	316	316	632
Cheul Ho Hong et al. (63)	2009	The Korean journal of gastroenterology	Korea	6	Colonoscopy & Biopsy	UC/CD	Histology	80	41	121
Garza- González et al. (32)	2010	International Journal of Immunogenetics	Mexico	7	Registry/Medical Record	CD/UC	Serology	44	75	119
Koskela et al. (64)	2011	Scandinavian Journal of Gastroenterology	Finland	7	Colonoscopy & Biopsy	IBDU	Histology	72	60	132
J. M. Thomson et al. (59)	2011	PLoS ONE	UK	6	Registry/Medical Record	UC	FISH	57	49	106
Zhang et al. (49)	2011	Journal of Clinical Microbiology	China	7	Colonoscopy & Biopsy	CD/UC	13C-UBT	208	416	624
Sonnenberg et al. (50)	2012	Alimentary Pharmacology and Therapeutics	USA	7	Registry/Medical Record	CD/UC/IBDU	Histology	1,064	64,451	65,515
Jin et al. (58)	2013	International Journal of Medical Sciences	China	7	Colonoscopy & Biopsy	UC	14C-UBT/histology	153	121	274
Xiang et al. (51)	2013	World Journal of Gastroenterology	China	6	Colonoscopy & Biopsy	CD	14C-UBT/Culture	229	248	477
M. Ram et al. (65)	2013	Clinical Chemistry and Laboratory Medicine	Europe	6	Registry/Medical Record	IBD	Serology	119	245	364
Magalhã es-Costa et al. (27)	2014	Arquivos de Gastroenterologia	Brazil	7	Colonoscopy & Biopsy	CD/UC	Histology	57	26	83
Farkas et al. (30)	2016	Journal of Crohn’s and Colitis	Hungary/ Hong Kong	6	Registry/Medical Record	CD	Histology	180	189	369
Mansour et al. (62)	2018	World Journal of Clinical Cases	Egypt	6	Registry/Medical Record	UC	Histology	30	30	60
Rosania et al. (55)	2018	Journal of Gastrointestinal and Liver Diseases	Germany	7	Registry/Medical Record	CD/UC	Serology	127	254	381
Sonnenberg et al. (54)	2018	Colorectal Disease	USA	6	Registry/Medical Record	CD/UC/IBDU	Histology	7,684	220,822	228,506
R. Sayar et al. (66)	2019	Caspian Journal of Internal Medicine	Iran	7	Colonoscopy & Biopsy	IBD	Serology	60	120	180
M. Varas Lorenzo et al. (56)	2019	Eurasian Journal of Medicine and Oncology	Spain	5	Registry/Medical Record	CD/UC/IBDU	13C-UBT	95	20	115
J. Ostrowski et al. (52)	2021	Scientific Reports	Poland	6	Registry/Medical Record	CD	RUT/Sequencing	24	19	43
Ding et al. (28)	2021	Plos One	China	7	Registry/Medical Record	CD/UC	Serology	260	520	780
Ali et al. (57)	2022	Heliyon	Palestine	7	Colonoscopy & Biopsy	UC	stool antigen test (SAT)	35	105	140
Graca-Pakulska et al. (8)	2023	Scientific reports	Poland	6	Registry/Medical Record	CD	RUT	62	199	261
Alotaibi et al. (53)	2025	BMC Gastroenterology	Saudi Arabia	7	Colonoscopy & Biopsy	CD/UC	stool antigen test (SAT)	180	180	360
Total								14,100	291,352	305,452

IBD, Inflammatory Bowel Diseases; Hp, Helicobacter pylori; Ag, Antigen; CD, Crohn’s Disease; UC, Ulcerative Colitis; IBDU, Inflammatory Bowel Diseases Unclassified; UBT, Urease Breath Test; RUT, Rapid Urease Test; PCR, Polymerase Chain Reaction; FISH, Fluorescence In Situ Hybridization.

### 3.3 Risk of bias assessment result

All included studies were subject to the risk of bias assessment with the Newcastle-Ottawa-Scale (NOS). The overall quality of the recruited studies was satisfactory, with 40 studies scoring over 6 stars (40/44, 90.91%) and half (22/44, 50.00%) scoring 7 stars or higher. When leave-one-out sensitivity analyses were conducted, all the results remained significant in both the overall and stratified analyses, indicating statistical robustness.

### 3.4 Association between *Helicobacter pylori* infection and IBD

**Figure 2:**
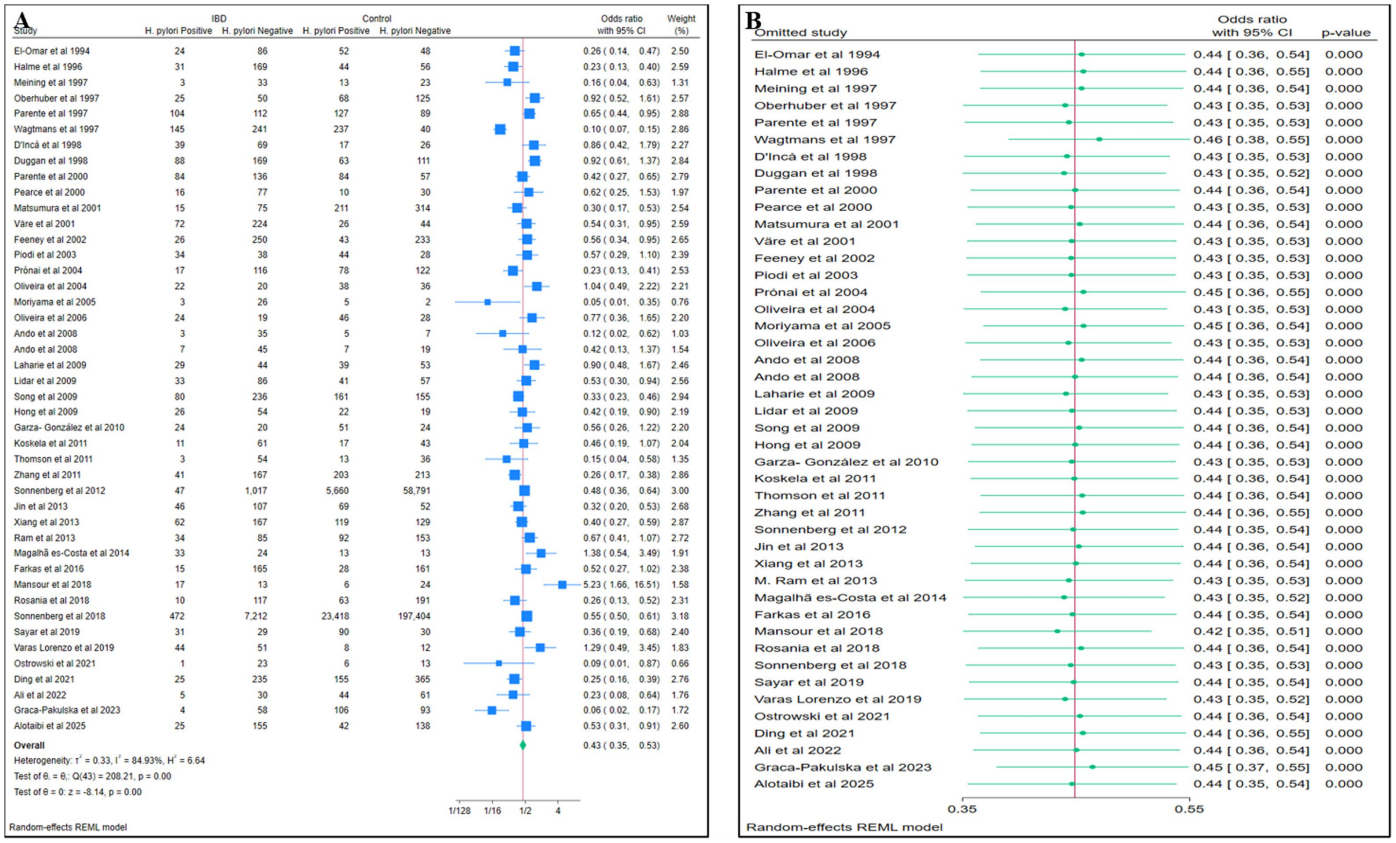
**(A)** Forest plots of the meta-analysis of *H. pylori* infection and IBD. **(B)** Leave-one-out sensitivity analysis.

The total study sample included 14,100 patients with IBD and 291,352 non-IBD controls. The population in most of the studies were middle-aged individuals. Data synthesis revealed a negative association between *H. pylori* infection and the condition of IBD (pooled OR:0.43, 95%CI: 0.35–0.53, *p* < 0.01; Figure 2A). Heterogeneity was high (I^2^ = 84.93%). The statistical robustness of the meta-analysis of *H. pylori* infection and IBD in the target population was proven with leave-one-out sensitivity analysis (Figure 2B).

### 3.5 Subgroup analyses

To explore potential sources of heterogeneity, we performed subgroup analyses across 9 dimensions, calculating pooled odds ratios (ORs) with 95% confidence intervals (CIs) and heterogeneity metrics (Cochrane’s Q, τ^2^, I^2^). Differences between subgroups were tested, and leave-one-out sensitivity analyses were conducted.

**Figure 3:**
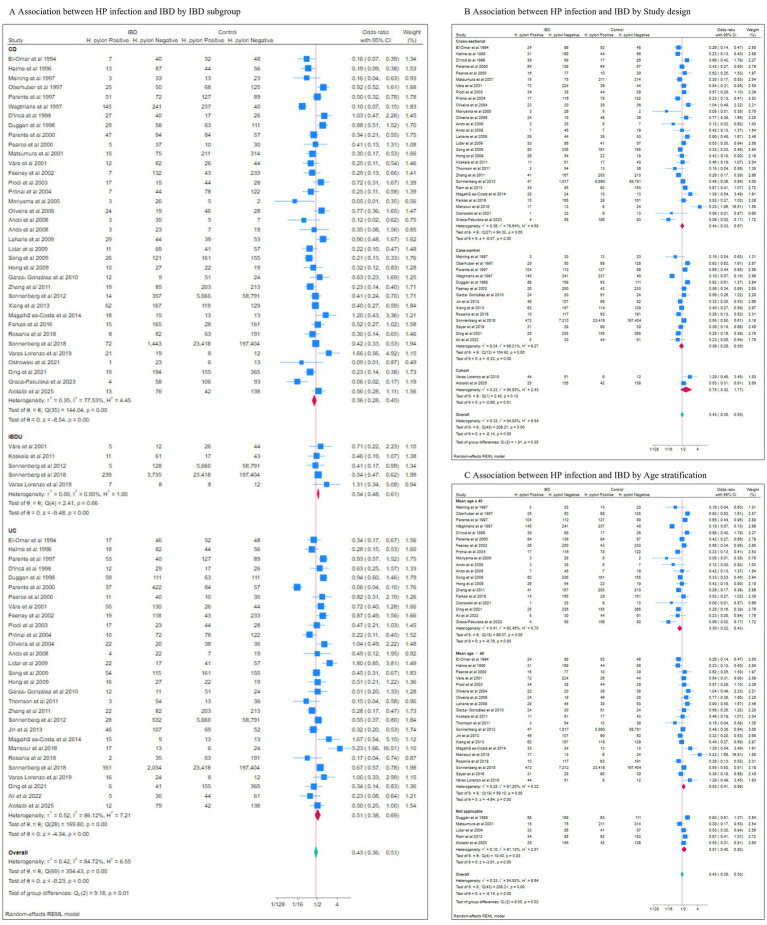
The results of different subgroup of analysis. **(A)** IBD subgroup; **(B)** Study design; **(C)** Age stratification.

A negative association between *H. pylori* infection and IBD was consistently observed across all subgroups (Figure 3; Table 2). This association was strongest in CD (pooled OR: 0.36, 95% CI: 0.28–0.45), followed by UC (pooled OR: 0.51, 95% CI: 0.38–0.69) and IBDU (pooled OR: 0.54, 95% CI: 0.48–0.61). Heterogeneity was high in the CD and UC subgroups but substantially lower in the IBDU subgroup.

**Table 2:** The results of different subgroup analysis.

Subgroups	No. of studies	No. of cases	No. of controls	Q	*p* value	τ^2^	I^2^(%)	OR 95%CI	*p* value	Test of group difference (P)
All studies	44	14,100	291,352	208.21	<0.01	0.33	84.93	0.43 (0.35,0.53)	<0.01	
IBD subtype										0.01
CD	36	4,954	290,549	144.04	<0.01	0.35	77.53	0.36 (0.28,0.45)	<0.01	
UC	29	4,756	289,056	169.80	<0.01	0.52	86.12	0.51 (0.38,0.69)	<0.01	
IBDU	5	4,211	285,423	2.41	0.66	0.00	0.00	0.54 (0.48,0.61)	<0.01	
Study design										0.38
Cross-sectional	28	3,987	67,715	94.32	<0.01	0.36	78.64	0.44 (0.33,0.57)	<0.01	
Case–control	14	9,838	223,437	104.92	<0.01	0.34	89.21	0.39 (0.28,0.55)	<0.01	
Cohort	2	275	200	2.43	0.12	0.23	58.83	0.75 (0.32,1.77)	0.51	
Age stratification										0.02
Mean age ≤ 40	19	2,734	3,232	96.07	<0.01	0.41	82.45	0.30 (0.22,0.43)	<0.01	
Mean age > 40	20	10,601	286,898	59.10	<0.01	0.25	81.20	0.53 (0.41,0.69)	<0.01	
Not applicable	5	765	1,222	10.40	0.03	0.10	61.12	0.43 (0.35,0.53)	<0.01	
Age difference										0.64
Age matched	35	13,435	290,532	164.42	<0.01	0.25	82.89	0.42 (0.34,0.51)	<0.01	
Control group is older	9	665	820	38.82	<0.01	0.86	85.25	0.50 (0.25,0.97)	0.04	
Ethnicity										0.08
Western	24	11,816	288,233	122.09	<0.01	0.28	85.00	0.46 (0.36,0.59)	<0.01	
Eastern	12	1931	2,482	15.66	0.15	0.01	16.19	0.34 (0.28,0.40)	<0.01	
Others	8	353	647	41.48	<0.01	1.53	87.57	0.57 (0.22,1.45)	0.24	
HP detection method										0.80
Serology	16	2,695	3,164	97.91	<0.01	0.32	81.16	0.42 (0.31,0.58)	<0.01	
Non-serology	28	11,405	288,188	100.99	<0.01	0.37	85.79	0.45 (0.34,0.59)	<0.01	
Definition of IBD										0.48
Registry/medical record	26	11,449	225,072	168.41	<0.01	0.53	90.76	0.42 (0.31,0.58)	<0.01	
Colonoscopy/Biopsy	14	2,332	66,044	29.51	<0.01	0.11	57.47	0.48 (0.38,0.62)	<0.01	
Not specified	4	319	236	4.83	0.18	0.11	37.30	0.34 (0.20,0.58)	<0.01	
Quality based on the risk of bias										0.24
High quality (≥7stars)	22	4,409	67,811	105.18	<0.01	0.25	78.60	0.39 (0.30,0.50)	<0.01	
Fair quality (≤6stars)	22	9,691	223,541	76.94	<0.01	0.45	86.09	0.50 (0.36,0.69)	<0.01	
Source of control										0.05
Healthy	19	2,802	2,937	93.59	<0.01	0.37	81.94	0.35 (0.25,0.48)	<0.01	
Other with-out IBD	25	11,298	288,415	84.97	<0.01	0.25	81.64	0.52 (0.41,0.66)	<0.01	

IBD, Inflammatory Bowel Diseases; CD, Crohn’s Disease; UC, Ulcerative Colitis; IBDU, Inflammatory Bowel Diseases Unclassified; OR, Odds Ratio; CI, Confidence Interval; HP, *Helicobacter pylori*.

Stratified analyses by study design, age stratification, age matching, ethnicity, *H. pylori* detection method, IBD definition, study quality and control source consistently revealed stable negative associations (Table 2). Notably, the association was significantly stronger in studies with younger populations (mean age ≤ 40; OR: 0.30, 95% CI: 0.22–0.43) than in those with older participants (mean age > 40; OR: 0.53, 95% CI: 0.41–0.69; *p* = 0.02 for subgroup difference). Study design did not significantly modify the effect (*p* = 0.38). The age-matched subgroup showed an effect size similar to the overall analysis (pooled OR: 0.42, 95% CI: 0.34–0.51, *p* < 0.01), with no significant between-subgroup difference (*p* = 0.64). Higher heterogeneity was observed in subgroups with older controls.

Negative association was observed in both Western (pooled OR: 0.46, 95% CI: 0.36–0.59, *p* < 0.01) and Eastern (pooled OR: 0.34, 95% CI: 0.28–0.40, *p* < 0.01) populations, but not in the “Others” category (pooled OR: 0.57, 95% CI: 0.22–1.45, *p* = 0.24). Heterogeneity was notably low in the Eastern subgroups.

**Figure 4:**
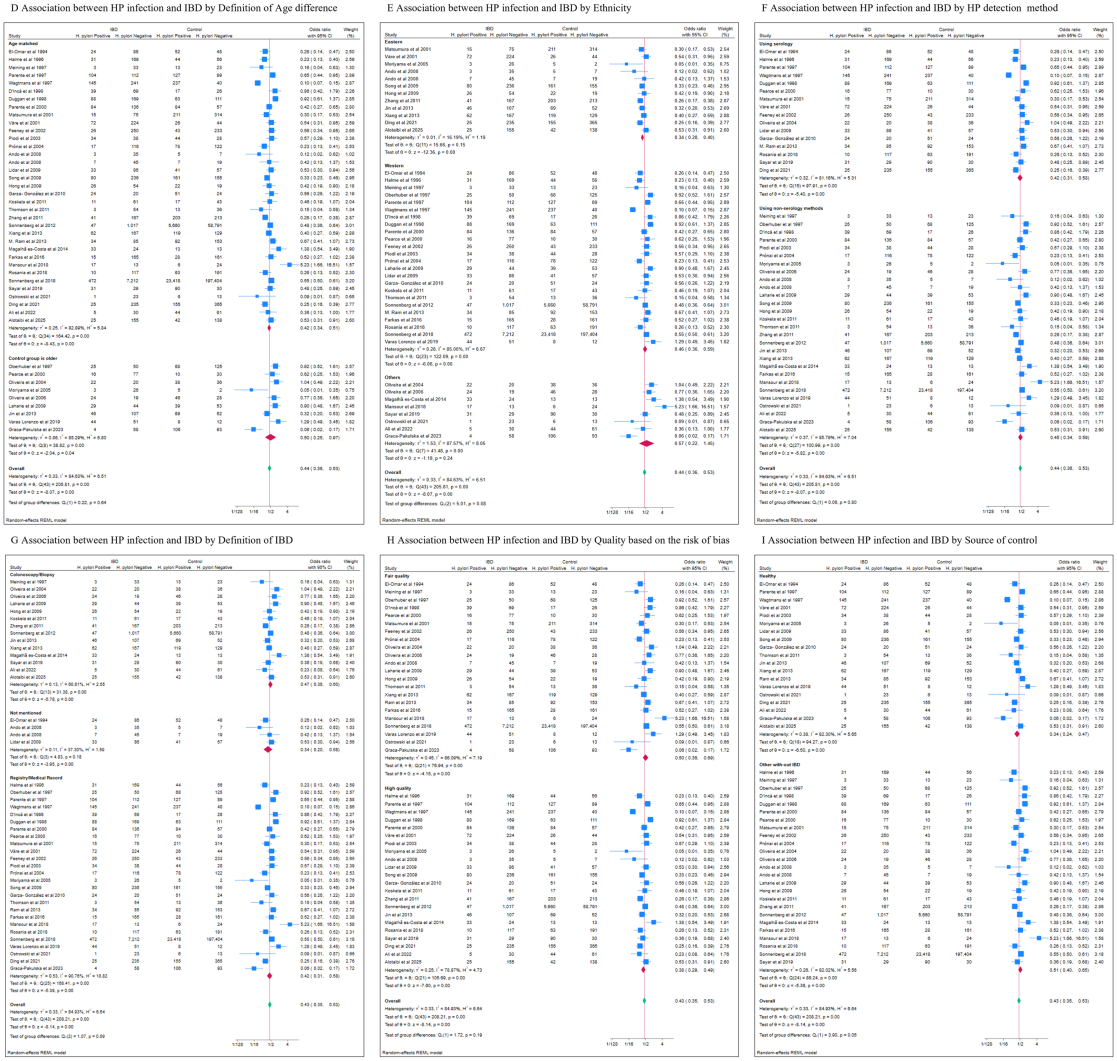
The results of different subgroup of analysis. **(D)** Age difference; **(E)** Ethnicity; **(F)** HP detection method; **(G)** Definition of IBD; **(H)** Quality based on the risk of bias; **(I)** Source of control.

When comparing serology-based detection of *H. pylori* with other methods, the serology subgroup exhibited a marginally stronger negative association (pooled OR: 0.42, 95% CI: 0.31–0.58, *p* < 0.01) than the non-serology subgroup (pooled OR: 0.45, 95% CI: 0.34–0.59, *p* < 0.01). Heterogeneity remained high in both, though sensitivity analyses confirmed result stability (Figure 4).

Most included studies defined IBD using either Registry/Hospital Record or Colonoscopy/Biopsy. Although the colonoscopy/biopsy subgroup was associated with lower heterogeneity, the effect sizes in both subgroups were comparable (Colonoscopy/Biopsy subgroup: pooled OR: 0.48, 95% CI: 0.38–0.62, *p* < 0.01; Registry/Medical Record subgroup: pooled OR: 0.42, 95% CI: 0.31–0.58, *p* < 0.01), with no statistically significant between-subgroup difference (*p* = 0.48).

A stronger negative association and lower heterogeneity level were observed in the high-quality subgroup (pooled OR: 0.39, 95% CI: 0.30–0.50, *p* < 0.01) compared to the fair-quality subgroup (pooled OR: 0.50, 95% CI: 0.36–0.69, *p* < 0.01). Similarly, studies using healthy controls demonstrated stronger negative association (pooled OR: 0.35, 95% CI: 0.25–0.48, *p* < 0.01) than those using non-IBD controls (pooled OR: 0.52, 95% CI: 0.41–0.66, *p* < 0.01), with a borderline significant subgroup difference (*p* = 0.05).

Despite extensive stratification, the definitive source of overall heterogeneity remained unexplained. Marked reductions in heterogeneity were observed in subgroups defined by Eastern ethnicity, IBDU diagnosis, colonoscopy-based IBD confirmation, and higher study quality. This pattern suggests that the observed heterogeneity stems from a combination of biological and methodological factors.

### 3.6 Meta-regression

Meta-regression analysis identified age stratification and control source as significant modifiers of the association between *H. pylori* infection and IBD: studies with participants aged > 40 years showed a significantly higher effect size (OR = 1.73, 95%CI: 1.13–2.62, *p* = 0.01), and those using non-IBD controls had a borderline significant higher effect size (OR = 1.48, 95%CI:0.99–2.21, *p* = 0.05). No significant differences were observed in other variables including IBD subtype, study design, ethnicity, and *H. pylori* detection method (Table 3).

**Table 3:** The results of meta-regression and publication bias assessment.

Subgroups	No. of studies	No. of cases	No. of controls	OR	95%CI	*p* value	Egger’s test p value
All studies	44	14,100	291,352				0.72
IBD subtype							
CD	36	4,954	290,549	0.69	0.48,1.00	0.53	0.69
UC	29	4,756	289,056		reference		0.82
IBDU	5	4,211	285,423	1.13	0.54,2.36	0.74	0.64
Study design							
Cross-sectional	28	3,987	67,715		reference		0.57
Case–control	14	9,838	223,437	0.09	0.58.1.39	0.64	0.16
Cohort	2	275	200	1.76	0.64,4.85	0.27	NA
Age stratification							
Mean age ≤ 40	19	2,734	3,232		reference		0.31
Mean age > 40	20	10,601	286,898	1.73	1.13,2.62	0.01	0.67
Not applicable	5	765	1,222	1.81	0.97,3.34	0.06	0.28
Age difference							
Age matched	35	13,435	290,532		Reference		0.66
Control group is older	9	665	820	1.27	0.76,2.13	0.36	0.13
Ethnicity							
Western	24	11,816	288,233	0.70	0.45,1.11	0.13	0.94
Eastern	12	1931	2,482		Reference		0.41
Others	8	353	647	1.31	0.73,2.33	0.36	0.54
HP detection method							
Serology	16	2,695	3,164		Reference		0.96
Non-serology	28	11,405	288,188	1.05	0.69,1.59	0.79	0.39
Definition of IBD							
Registry/medical record	26	11,449	225,072	1.16	0.74,1.81	0.52	0.59
Colonoscopy/Biopsy	14	2,332	66,044		Reference		0.86
Not specified	4	319	236	0.77	0.35,1.69	0.51	0.49
Quality based on the risk of bias							
High quality (≥7stars)	22	4,409	67,811	1.29	0.87,1.93	0.20	0.26
Fair quality (≤6stars)	22	9,691	223,541		Reference		0.54
Source of control							
Healthy	19	2,802	2,937		Reference		0.65
Other with-out IBD	25	11,298	288,415	1.48	0.99,2.21	0.05	0.20

IBD, Inflammatory Bowel Diseases; CD, Crohn’s Disease; UC, Ulcerative Colitis; IBDU, Inflammatory Bowel Diseases Unclassified; OR, Odds Ratio; CI, Confidence Interval; HP, *Helicobacter pylori*.

### 3.7 Assessment of publication bias

**Figure 5:**
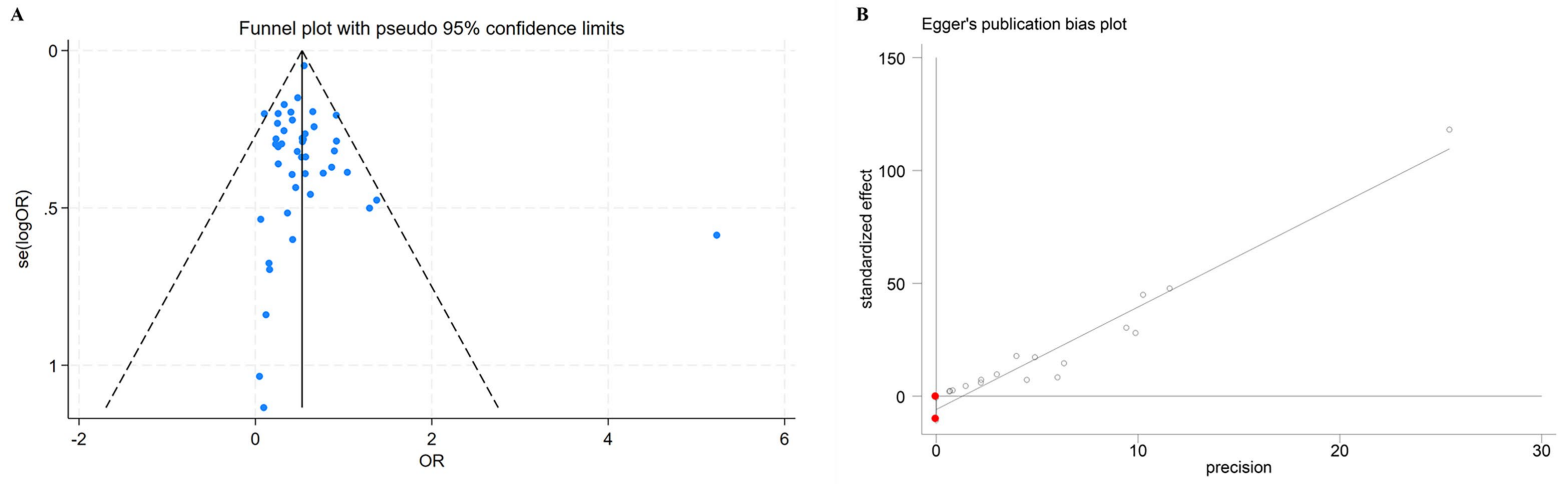
**(A)** Assessment of publication bias using funnel plots. Each dot represents one study. **(B)** Egger’s tests were used to verify the possibility of publication bias. The distance between the two red dots on the ordinate represents the 95% confidence interval.

Publication bias was assessed for the overall set of included studies and for each subgroup. Although the funnel plot appeared slightly asymmetric (Figure 5), Egger’s test revealed no statistically significant evidence of publication bias in the overall analysis (*p* = 0.72) or in any of the subgroup analyses (Table 3).

### 3.8 Sensitivity analysis

Sensitivity analyses confirmed the robustness of the primary findings. Exclusion of 22 lower-quality studies (NOS < 7) yielded a pooled OR of 0.38 (95% CI: 0.29–0.49, *p* < 0.01) with reduced heterogeneity (I^2^ = 78.87%). Similarly, excluding 7 studies with sample sizes < 100 (OR: 0.43, 95% CI: 0.35–0.52) or 5 studies with unclear age stratification (“Not Applicable”; OR: 0.41, 95% CI: 0.33–0.52) did not alter the overall conclusion (Supplementary Figures 2–4).

## 4 Discussion

The current meta-analysis pooled data from 44 original studies published up to July 10th,2025, from 21 countries. The total number of IBD patients and controls were 14,100 and 291,352, respectively. Data synthesis using a random-effect model revealed a significant reduction in the odds of IBD among individuals infected with *H. pylori* (pooled OR: 0.43, 95%CI: 0.35–0.53, *p* < 0.01), particularly in CD (pooled OR: 0.36, 95% CI: 0.28–0.45, *p* < 0.01) and the Eastern population (pooled OR: 0.34, 95% CI: 0.28–0.40, *p* < 0.01). These results suggested a potential protective effect associated with the pathogen.

*H. pylori* was negatively associated with several atopic and inflammatory diseases, such as asthma and eczema (67, 68). Hypothesis was that the association may also exist in other Regulatory T cell (Treg)-associated autoimmune diseases. *H. pylori*’s immunomodulatory effect was modulated by Interleukin (IL)-18-producing, tolerogenic dendritic cells, which were able to induce T-cell conversion toward Foxp3 + Tregs and control the degree of inflammation (69). Recently, the cytotoxin-associated gene A (CagA), an important virulence factor of *H. pylori* typically associated with an increased risk of gastric cancer, was also found to have immunomodulatory effect. The meta-analysis by Tepler et al. found that serologic response to CagA was related to substantially lower odds of IBD and that individuals exposed to CagA-negative H.pylori were associated with similar odds of IBD to individuals without any exposure to the pathogen, supporting the hypothesis that CagA might be a key determinant in the protective association conferred by *H. pylori* (14).

The potential protective effect of *H. pylori* may also be related to the preservation of a healthy gut microbiome. *H. pylori* may alter gut microbiota and reduce dysbiosis linked to IBD, which typically shows less diversity and abundance than healthy individuals (70). This alteration also reduces the risk of pathogenic bacterial overgrowth and the subsequent immune response leading to chronic intestinal inflammation. However, a direct causal relationship between dysbiosis and IBD has not been definitively established in humans (71). A meta-analysis by Zhong et al. noted a higher recurrence rate of inflammatory bowel disease following *Helicobacter pylori* eradication, suggesting a potential link to gut microbiota dysbiosis (18).

To explore the sources of the substantial observed heterogeneity (I^2^ = 84.93%), subgroup analyses were performed based on pre-specified biological and methodological factors. The subgroup analysis stratified data according to 9 biological or methodological factors. Among these, IBD subtype, age stratification and source of control were associated with significant differences between the subgroups. The negative association between *H. pylori* and IBD was stronger in CD compared to UC and IBDU, and in the Eastern population compared to the Western population, corroborating previous studies (9, 16).

The differences in the disease subtypes could be explained by the immunologic profiles of the two conditions. The pathogenesis of IBD includes an inappropriate immune response generated by the genetically susceptible host to the intestinal microbiome (72, 73). Despite both being a mixed lymphocyte reaction involving Th1, Th2, Th9, Th17, and Treg, CD is characterized by a stronger Th1 response with IL-23/Th17 activation, in comparison to UC, which is predominantly a Th2-like response characterized by increased IL-13 and IL-5 (74, 75).

The difference in CagA expression between Western and Eastern *H. pylori* strains is possibly another explanation for the ethnic disparity in the result. Nearly 100% of the Eastern strain of *H. pylori* was found expressing CagA, compared to only 60–70% of the Western strain (76). Therefore, the Eastern population infected by *H. pylori* may develop a stronger immunomodulatory response with a CagA-positive strain.

Environmental and socioeconomic factors, such as diet and childhood environment, also contribute to the pathogenesis of IBD. The Western diet characterized by lower fiber and higher refined carbohydrates and processed meat is considered conducive to IBD (77). The incidence rates of IBD have been steadily rising in rapidly developing countries where changes in the structure of diet toward the Western style are happening (2). Epidemiological studies conducted on the first and second generations of immigrants from Asia, Latin America, Africa, and the Middle East to Canada found that second-generation immigrants shared a similar incidence rate of IBD with native Canadian children, which was much higher than that in their countries of origin (78).

The more pronounced negative association in younger populations (mean age ≤ 40 years; OR = 0.30) compared to older groups (OR = 0.53) may reflect age-dependent immune responsiveness. *H. pylori* infection typically occurs in childhood, and early-life exposure may induce long-term immunomodulatory effects (69) that are more effective in preventing the development of IBD, which often onset in early adulthood (14). In older individuals, cumulative environmental exposures, comorbidities, and age-related immune senescence may weaken the protective effect of *H. pylori* (79). Additionally, older populations may have a higher prevalence of prior *H. pylori* eradication therapy or spontaneous clearance, which could confound the association (80).

On a methodological level, several factors jointly contribute to the variation in effect sizes. Firstly, the diagnostic methods for defining *H. pylori* status were not uniform. Serology defines an “exposed” population that differs from those defined by tests for active infection, such as the urea breath test and histology. Secondly, the source of the control group significantly influenced the effect size, with studies using healthy controls demonstrating a stronger inverse association. This phenomenon is likely attributable to Berkson’s bias (81). Non-IBD patients presenting with gastrointestinal symptoms have an inherently higher prevalence of *H. pylori* infection than the general healthy population. Using this inflated baseline for comparison artificially diminishes the observed difference in infection rates between cases and controls. Thirdly, the effect sizes were similar regardless of whether IBD was defined by colonoscopy/biopsy or registry/medical records (*p* = 0.48). However, the subgroup using colonoscopy/biopsy was associated with a lower level of heterogeneity, suggesting that more stringent diagnostic criteria contribute to greater consistency in results. Finally, and most critically, many original studies failed to adequately control for or report key confounding variables, such as the use of 5-aminosalicylates commonly prescribed to IBD patients, a history of prior *H. pylori* eradication therapy, and disease activity at the time of testing. These factors could introduce bias and lead to inconsistencies between studies. Consequently, the pooled effect size presented in this meta-analysis should be interpreted as an overall estimate under the joint influence of these biological and methodological factors. The preceding subgroup analyses were instrumental in deconstructing this complexity and confirming the robustness of the core finding across various contexts.

Being a multifactorial disease, the study of IBD benefits from a large pooled sample size and the inclusion of recent studies. This is the 13th meta-analysis focusing on *H. pylori* and IBD, and the 9th specifically analyzing the association between *H. pylori* infection and IBD onset (9–19). Luther et al. (12), in their 2010 study of 5,903 subjects, first reported a statistically significant association between *H. pylori* and IBD (RR: 0.64, 95%CI:0.54–0.75). This finding was later confirmed by Wu et al. (16)(RR: 0.48, 95%CI:0.43–0.54) and Rokkas et al. (19) (RR: 0.62, 95%CI:0.55–0.71) in 2015. Castaño-Rodríguez et al. (9) not only further validated this negative association (P-OR: 0.43, 95%CI:0.36–0.50), but also verified its robustness across different ethnicities, age groups, and detection methods of H pylori. Recently, Shirzad-Aski et al. conducted a study with the largest pooled sample size (13,549 individuals from 58 studies up to June 2018), which yielded similar results (13). However, in that meta-analysis, two studies conducted by Sonnenberg et al. using the same electronic database with another two in an overlapping time frame were included, raising concerns about data redundancy (50). These studies were excluded in this meta-analysis (22, 23). Compared to the 2020 study by Shirzad-Aski, the current meta-analysis included some of the latest original researches up to July 10th, 2025. Only published resources were sought, and statistical results from conference proceedings, comments, letters, and editorials were excluded for greater statistical robustness. With a stricter inclusion and exclusion criteria and statistical rigor, the current meta-analysis offers a reliable update and extension of the preceding works.

Despite the strengths of this meta-analysis stemming from the comprehensive literature search, rigorous methodological approach, and large sample size, there are still limitations to consider. First, the high heterogeneity among the included studies, which was not effectively addressed by any subgroup analyses, may have introduced bias. Second, in pursuit of statistical robustness, the current meta-analysis sought only published data in peer-reviewed publications, which inevitably lowered the scale of the total sample size. Third, our literature search was restricted to English-language publications. While this ensured accuracy in data handling, it may have introduced language bias by excluding relevant studies published in other languages, particularly from Eastern regions. This could potentially affect the generalizability of our findings. However, the consistent negative association observed specifically within the Eastern subgroup, derived from a substantial number of English-language studies conducted in those regions, suggests that the core finding remains robust. Moreover, most observational studies not containing sufficient background data during their publication had limited further exploration of heterogeneity sources. These missing data included but were not limited to: the exact timing of pylori testing in relation to the diagnosis of IBD, the history of *H. pylori* eradication, the extent and severity of IBD according to the Montreal Classification, and the detailed medication history. However, it should be acknowledged that since the investigation on the relationship between *H. pylori* and IBD has spanned nearly 3 decades and the concepts and therapeutic techniques have been constantly evolving, setting too high a bar for certain previous studies is unreasonable. Hopefully, future studies will document or control for these factors, helping elucidate the true nature of the relationship between *H. pylori* infection and IBD. Besides, despite being statistically insignificant, the effect of publication bias should not be neglected. Finally, although this meta-analysis confirms a negative association between *H. pylori* infection and IBD, as with all epidemiological studies, this association does not necessarily imply causality and should be interpreted cautiously.

In conclusion, this meta-analysis demonstrates a significant negative association between *H. pylori* infection and IBD, particularly in patients with Crohn’s disease, younger populations and Eastern populations. This specific pattern of association—most pronounced in a condition dominated by Th1/Th17 immune responses (CD) and among populations with a high prevalence of CagA-positive strains—suggests that *H. pylori*, particularly CagA-positive strains, may play a potential protective role, likely mediated through immunomodulatory mechanisms. However, it is crucial to emphasize that this finding is based on observational evidence and cannot establish causality. Current clinical guidelines recommending *H. pylori* eradication for gastric health remain largely applicable. The decision to eradicate *H. pylori* in patients with IBD should be individualized, based on a comprehensive assessment of factors such as gastric cancer risk, symptomatology, and disease activity. Therefore, the management of *H. pylori* infection in this population should shift from a routine eradication approach toward a more prudent and individualized strategy.

## Data Availability

The original contributions presented in the study are included in the article/Supplementary material, further inquiries can be directed to the corresponding author.

## Supplementary material

SUPPLEMENTARY TABLE 1 Search Syntax of the Databases.

SUPPLEMENTARY IMAGE 1 Association between Hp infection and IBD stratified by subgroup.

SUPPLEMENTARY IMAGE 2 Results of sensitivity analyses, excluded lower-quality studies.

SUPPLEMENTARY IMAGE 3 Results of sensitivity analyses excluded studies with a sample size smaller than 100.

SUPPLEMENTARY IMAGE 4 Results of sensitivity analyses excluded studies with unclear age stratification.

## References

[1] Guan Q. A comprehensive review and update on the pathogenesis of inflammatory bowel disease. J Immunol Res. (2019) 2019:7247238. doi: 10.1155/2019/7247238, 31886308 PMC6914932

[2] Wang R Li Z Liu S Zhang D. Global, regional and national burden of inflammatory bowel disease in 204 countries and territories from 1990 to 2019: a systematic analysis based on the global burden of disease study 2019. BMJ Open. (2023) 13:e065186. doi: 10.1136/bmjopen-2022-065186, 36977543 PMC10069527

[3] Lewis JD Parlett LE Jonsson Funk ML Brensinger C Pate V Wu Q . Incidence, prevalence, and racial and ethnic distribution of inflammatory bowel disease in the United States. Gastroenterology. (2023) 165:1197–1205.e2. doi: 10.1053/j.gastro.2023.07.003, 37481117 PMC10592313

[4] Kusters JG van Vliet AH Kuipers EJ. Pathogenesis of *Helicobacter pylori* infection. Clin Microbiol Rev. (2006) 19:449–90. doi: 10.1128/CMR.00054-05, 16847081 PMC1539101

[5] Sugano K Tack J Kuipers EJ Graham DY el-Omar EM Miura S . Kyoto global consensus report on *Helicobacter pylori* gastritis. Gut. (2015) 64:1353–67. doi: 10.1136/gutjnl-2015-309252, 26187502 PMC4552923

[6] Ormand JE Talley NJ Shorter RG. Prevalence of *Helicobacter pylori* in specific forms of gastritis. Further evidence supporting a pathogenic role for *H. pylori* in chronic nonspecific gastritis. Dig Dis Sci. (1991) 36:142–5. 1988256 10.1007/BF01300747

[7] El-Omar E Penman I Cruikshank G Dover S Banerjee S Williams C . Low prevalence of *Helicobacter pylori* in inflammatory bowel disease: association with sulphasalazine. Gut. (1994) 35:1385–8. 7959192 10.1136/gut.35.10.1385 PMC1375010

[8] Graca-Pakulska K Błogowski W Zawada I Deskur A Dąbkowski K Urasińska E . Endoscopic findings in the upper gastrointestinal tract in patients with Crohn's disease are common, highly specific, and associated with chronic gastritis. Sci Rep. (2023) 13:703. doi: 10.1038/s41598-022-21630-5, 36639398 PMC9839771

[9] Castaño-Rodríguez N Kaakoush NO Lee WS Mitchell HM. Dual role of Helicobacter and Campylobacter species in IBD: a systematic review and meta-analysis. Gut. (2017) 66:235–49. doi: 10.1136/gutjnl-2015-310545, 26508508

[10] Imawana RA Smith DR Goodson ML. The relationship between inflammatory bowel disease and *Helicobacter pylori* across east Asian, European and Mediterranean countries: a meta-analysis. Ann Gastroenterol. (2020) 33:485–94. doi: 10.20524/aog.2020.0507, 32879595 PMC7406810

[11] Kong G Liu Z Lu Y Li M Guo H. The association between *Helicobacter pylori* infection and inflammatory bowel disease in children: a systematic review with meta-analysis. Medicine (Baltimore). (2023) 102:e34882. doi: 10.1097/MD.0000000000034882, 37682136 PMC10489354

[12] Luther J Dave M Higgins PD Kao JY. Association between *Helicobacter pylori* infection and inflammatory bowel disease: a meta-analysis and systematic review of the literature. Inflamm Bowel Dis. (2010) 16:1077–84. doi: 10.1002/ibd.21116, 19760778 PMC4865406

[13] Shirzad-Aski H Besharat S Kienesberger S Sohrabi A Roshandel G Amiriani T . Association between *Helicobacter pylori* colonization and inflammatory bowel disease: a systematic review and Meta-analysis. J Clin Gastroenterol. (2021) 55:380–92. doi: 10.1097/MCG.0000000000001415, 32833699

[14] Tepler A Narula N Peek RM Jr Patel A Edelson C Colombel JF . Systematic review with meta-analysis: association between *Helicobacter pylori* CagA seropositivity and odds of inflammatory bowel disease. Aliment Pharmacol Ther. (2019) 50:121–31. doi: 10.1111/apt.15306, 31165513 PMC7393806

[15] Wang WL Xu XJ. Correlation between *Helicobacter pylori* infection and Crohn's disease: a meta-analysis. Eur Rev Med Pharmacol Sci. (2019) 23:10509–16. doi: 10.26355/eurrev_201912_19691, 31841206

[16] Wu XW Ji HZ Yang MF Wu L Wang FY. *Helicobacter pylori* infection and inflammatory bowel disease in Asians: a meta-analysis. World J Gastroenterol. (2015) 21:4750–6. doi: 10.3748/wjg.v21.i15.4750, 25914487 PMC4402325

[17] Yu Q Zhang S Li L Xiong L Chao K Zhong B . Enterohepatic Helicobacter species as a potential causative factor in inflammatory bowel disease: a Meta-analysis. Medicine (Baltimore). (2015) 94:e1773. doi: 10.1097/MD.0000000000001773, 26559250 PMC4912244

[18] Zhong Y Zhang Z Lin Y Wu L. The relationship between *Helicobacter pylori* and inflammatory bowel disease. Arch Iran Med. (2021) 24:317–25. doi: 10.34172/aim.2021.44, 34196192

[19] Rokkas T Gisbert JP Niv Y O'Morain C. The association between *Helicobacter pylori* infection and inflammatory bowel disease based on meta-analysis. United European Gastroenterol J. (2015) 3:539–50. doi: 10.1177/2050640615580889, 26668747 PMC4669512

[20] Page MJ McKenzie JE Bossuyt PM. The PRISMA 2020 statement: an updated guideline for reporting systematic reviews. BMJ. (2021) 372:n71. doi: 10.1136/bmj.n71 33782057 PMC8005924

[21] Higgins JP Thompson SG Deeks JJ Altman DG. Measuring inconsistency in meta-analyses. BMJ. (2003) 327:557–60. doi: 10.1136/bmj.327.7414.557, 12958120 PMC192859

[22] Sonnenberg A Genta RM. Inverse association between *Helicobacter pylori* gastritis and microscopic colitis. Inflamm Bowel Dis. (2016) 22:182–6. doi: 10.1097/MIB.0000000000000595, 26383914

[23] Sonnenberg A Melton SD Genta RM. Frequent occurrence of gastritis and Duodenitis in patients with inflammatory bowel disease. Inflamm Bowel Dis. (2011) 17:39–44. doi: 10.1002/ibd.21356, 20848539

[24] Ando T Watanabe O Ishiguro K Maeda O Ishikawa D Minami M . Relationships between *Helicobacter pylori* infection status, endoscopic, histopathological findings, and cytokine production in the duodenum of Crohn's disease patients. J Gastroenterol Hepatol. (2008) 23:S193–7. doi: 10.1111/j.1440-1746.2008.05438.x 19120897

[25] Basset C Holton J Bazeos A Vaira D Bloom S. Are *Helicobacter* species and enterotoxigenic *Bacteroides fragilis* involved in inflammatory bowel disease? Dig Dis Sci. (2004) 49:1425–32. doi: 10.1023/b:ddas.0000042241.13489.88, 15481314

[26] D'Incà R Sturniolo G Cassaro M di Pace C Longo G Callegari I . Prevalence of upper gastrointestinal lesions and *Helicobacter pylori* infection in Crohn's disease. Dig Dis Sci. (1998) 43:988–92. 9590412 10.1023/a:1018870415898

[27] Magalhães-Costa MH Reis BR Chagas VL Nunes T Souza HS Zaltman C. Focal enhanced gastritis and macrophage microaggregates in the gastric mucosa: potential role in the differential diagnosis between Crohn's disease and ulcerative colitis. Arq Gastroenterol. (2014) 51:276–82. doi: 10.1590/S0004-28032014000400003, 25591154

[28] Ding ZH Xu XP Wang TR Liang X Ran ZH Lu H. The prevalence of *Helicobacter pylori* infection in inflammatory bowel disease in China: a case-control study. PLoS One. (2021) 16:e0248427. doi: 10.1371/journal.pone.0248427, 33711050 PMC7954320

[29] Duggan AE Usmani I Neal KR Logan RF. Appendicectomy, childhood hygiene, *Helicobacter pylori* status, and risk of inflammatory bowel disease: a case control study. Gut. (1998) 43:494–8. 9824576 10.1136/gut.43.4.494 PMC1727261

[30] Farkas K Chan H Rutka M Szepes Z Nagy F Tiszlavicz L . Gastroduodenal involvement in asymptomatic Crohn's disease patients in two areas of emerging disease: Asia and Eastern Europe. J Crohns Colitis. (2016) 10:1401–6. doi: 10.1093/ecco-jcc/jjw113, 27282400

[31] Feeney MA Murphy F Clegg AJ Trebble TM Sharer NM Snook JA. A case-control study of childhood environmental risk factors for the development of inflammatory bowel disease. Eur J Gastroenterol Hepatol. (2002) 14:529–34. doi: 10.1097/00042737-200205000-00010, 11984151

[32] Garza-Gonzalez E Perez-Perez GI Mendoza-Ibarra SI Flores-Gutiérrez JP Bosques-Padilla FJ. Genetic risk factors for inflammatory bowel disease in a north-eastern Mexican population. Int J Immunogenet. (2010) 37:355–9. doi: 10.1111/j.1744-313X.2010.00932.x, 20518842

[33] Halme L Rautelin H Leidenius M Kosunen TU. Inverse correlation between *Helicobacter pylori* infection and inflammatory bowel disease. J Clin Pathol. (1996) 49:65–7. 8666689 10.1136/jcp.49.1.65 PMC1023160

[34] Meining A Bayerdörffer E Bastlein E Bastlein E Raudis N Thiede C . Focal inflammatory infiltrations in gastric biopsy specimens are suggestive of Crohn's disease. Crohn's Dis Study Group, Germany Scand J Gastroenterol. (1997) 32:813–8. 10.3109/00365529708996539 9282974

[35] Oberhuber G Püspök A Oesterreicher C Novacek G Zauner C Burghuber M . Focally enhanced gastritis: a frequent type of gastritis in patients with Crohn's disease. Gastroenterology. (1997) 112:698–706. doi: 10.1053/gast.1997.v112.pm9041230, 9041230

[36] Wagtmans MJ Witte AM Taylor DR Biemond I Veenendaal RA Verspaget HW . Low seroprevalence of *Helicobacter pylori* antibodies in historical sera of patients with Crohn's disease. Scand J Gastroenterol. (1997) 32:712–8. doi: 10.3109/00365529708996523, 9246713

[37] Parente F Cucino C Bollani S Imbesi V Maconi G Bonetto S . Focal gastric inflammatory infiltrates in inflammatory bowel diseases: prevalence, immunohistochemical characteristics, and diagnostic role. Am J Gastroenterol. (2000) 95:705–11. doi: 10.1111/j.1572-0241.2000.01851.x, 10710061

[38] Pearce CB Duncan HD Timmis L Green JR. Assessment of the prevalence of infection with *Helicobacter pylori* in patients with inflammatory bowel disease. Eur J Gastroenterol Hepatol. (2000) 12:439–43. 10783998 10.1097/00042737-200012040-00012

[39] Matsumura M Matsui T Hatakeyama S Matake H Uno H Sakurai T . Prevalence of *Helicobacter pylori* infection and correlation between severity of upper gastrointestinal lesions and *H. pylori* infection in Japanese patients with Crohn's disease. J Gastroenterol. (2001) 36:740–7. doi: 10.1007/s005350170015 11757745

[40] Väre PO Heikius B Silvennoinen JA Karttunen R Niemelä SE Lehtola JK . Seroprevalence of *Helicobacter pylori* infection in inflammatory bowel disease: is *Helicobacter pylori* infection a protective factor? Scand J Gastroenterol. (2001) 36:1295–300. doi: 10.1080/003655201317097155, 11761020

[41] Piodi LP Bardella M Rocchia C Cesana BM Baldassarri A Quatrini M. Possible protective effect of 5-aminosalicylic acid on *Helicobacter pylori* infection in patients with inflammatory bowel disease. J Clin Gastroenterol. (2003) 36:22–5. doi: 10.1097/00004836-200301000-00008, 12488702

[42] Prónai L Schandl L Orosz Z Magyar P Tulassay Z. Lower prevalence of *Helicobacter pylori* infection in patients with inflammatory bowel disease but not with chronic obstructive pulmonary disease - antibiotic use in the history does not play a significant role. Helicobacter. (2004) 9:278–83. doi: 10.1111/j.1083-4389.2004.00223.x, 15165265

[43] Moriyama T Matsumoto T Jo Y Yada S Hirahashi M Yao T . Mucosal proinflammatory cytokine and chemokine expression of gastroduodenal lesions in Crohn's disease. Aliment Pharmacol Ther. (2005) 21:85–91. doi: 10.1111/j.1365-2036.2005.02480.x 15943853

[44] Oliveira AG Rocha GA Rocha AM Sanna Md Moura SB Dani R . Isolation of *Helicobacter pylori* from the intestinal mucosa of patients with Crohn's disease. Helicobacter. (2006) 11:2–9. doi: 10.1111/j.0083-8703.2006.00368.x 16423084

[45] Ando T Watanabe O Nobata K Ishiguro K Maeda O Ishikawa D . Immunological status of the stomach in inflammatory bowel disease - comparison between ulcerative colitis and Crohn's disease. Digestion. (2008) 77:145–9. doi: 10.1159/000140973, 18577851

[46] Laharie D Asencio C Asselineau J Bulois P Bourreille A Moreau J . Association between entero-hepatic Helicobacter species and Crohn's disease: a prospective cross-sectional study. Aliment Pharmacol Ther. (2009) 30:283–93. doi: 10.1111/j.1365-2036.2009.04034.x, 19438427

[47] Lidar M Langevitz P Barzilai O Ram M Porat-Katz BS Bizzaro N . Infectious serologies and autoantibodies in inflammatory bowel disease: insinuations at a true pathogenic role. Ann N Y Acad Sci. (2009) 1173:640–8. doi: 10.1111/j.1749-6632.2009.04673.x, 19758210

[48] Song MJ Park DI Hwang SJ Kim ER Kim YH Jang BI . The prevalence of *Helicobacter pylori* infection in Korean patients with inflammatory bowel disease, a multicenter study. Korean J Gastroenterol. (2009) 53:341–7. doi: 10.4166/kjg.2009.53.6.341, 19556840

[49] Zhang S Zhong B Chao K. Role of Helicobacter species in Chinese patients with inflammatory bowel disease. J Clin Microbiol. (2011) 49:1987–9. doi: 10.1128/JCM.02630-10, 21346040 PMC3122638

[50] Sonnenberg A Genta RM. Low prevalence of *Helicobacter pylori* infection among patients with inflammatory bowel disease. Aliment Pharmacol Ther. (2012) 35:469–76. doi: 10.1111/j.1365-2036.2011.04969.x, 22221289

[51] Xiang Z Chen YP Ye YF Ma KF Chen SH Zheng L . Helicobacter pylori and Crohn's disease: a retrospective single-center study from China. World J Gastroenterol. (2013) 19:4576–81. doi: 10.3748/wjg.v19.i28.4576, 23901235 PMC3725384

[52] Ostrowski J Kulecka M Zawada I Żeber-Lubecka N Paziewska A Graca-Pakulska K. The gastric microbiota in patients with Crohn's disease; a preliminary study. Sci Rep. (2021) 11:17866. doi: 10.1038/s41598-021-97261-z, 34504159 PMC8429686

[53] Alotaibi AD Al-Abdulwahab AA Ismail MH. Prevalence of *H. pylori* in inflammatory bowel disease patients and its association with severity. BMC Gastroenterol. (2025) 25:892. doi: 10.1186/s12876-025-03892-1 40301738 PMC12042615

[54] Sonnenberg A Turner KO Genta RM. Decreased risk for microscopic colitis and inflammatory bowel disease among patients with reflux disease. Color Dis. (2018) 20:813–20. doi: 10.1111/codi.14114, 29603881

[55] Rosania R Von Arnim U Link A Rajilic-Stojanovic M Franck C Canbay A . *Helicobacter pylori* eradication therapy is not associated with the onset of inflammatory bowel diseases. A case-control study. J Gastrointestin Liver Dis. (2018) 27:119–25. doi: 10.15403/jgld.2014.1121.272.hpy, 29922755

[56] Lorenzo MV Agel FM Mengual ES-V. Eradication of *Helicobacter pylori* in patients with inflammatory bowel disease for prevention of recurrences - impact on the natural history of the disease. Eurasian J Med Oncol. (2019). 3:59–65. doi: 10.14744/ejmo.2018.0058

[57] Ali I Abdo Q Al-Hihi S. Association between ulcerative colitis and *Helicobacter pylori* infection: a case-control study. Heliyon. (2022) 8:e08930. doi: 10.1016/j.heliyon.2022.e08930 35198786 PMC8841358

[58] Jin X Chen YP Chen SH Xiang Z. Association between *Helicobacter Pylori* infection and ulcerative colitis--a case control study from China. Int J Med Sci. (2013) 10:1479–84. doi: 10.7150/ijms.6934, 24046521 PMC3775104

[59] Thomson JM Hansen R Berry SH Hope ME Murray GI Mukhopadhya I . Enterohepatic helicobacter in ulcerative colitis: potential pathogenic entities? PLoS One. (2011) 6:e17184. doi: 10.1371/journal.pone.0017184, 21383845 PMC3044171

[60] Oliveira AG das Graças Pimenta Sanna M Rocha GA Rocha AM Santos A Dani R . Helicobacter species in the intestinal mucosa of patients with ulcerative colitis. J Clin Microbiol. (2004) 42:384–6. doi: 10.1128/JCM.42.1.384-386.2004 14715785 PMC321729

[61] Parente F Molteni P Bollani S Maconi G Vago L Duca PG . Prevalence of *Helicobacter pylori* infection and related upper gastrointestinal lesions in patients with inflammatory bowel diseases. A cross-sectional study with matching. Scand J Gastroenterol. (1997) 32:1140–6. doi: 10.3109/00365529709002994 9399396

[62] Mansour L El-Kalla F Kobtan A Abd-Elsalam S Yousef M Soliman S . *Helicobacter pylori* may be an initiating factor in newly diagnosed ulcerative colitis patients: a pilot study. World J Clin Cases. (2018) 6:641–9. doi: 10.12998/wjcc.v6.i13.641, 30430119 PMC6232561

[63] Cheul Ho Hong DIP Choi WH Park JH Kim HJ Cho YK. The clinical usefulness of focally enhanced gastritis in Korean patients with Crohn’s disease. Korean J Gastroenterol. (2009). 53:23–8. 19158467

[64] Koskela RM Niemelä SE Lehtola JK Bloigu RS Karttunen TJ. Gastroduodenal mucosa in microscopic colitis. Scand J Gastroenterol. (2011) 46:567–76. doi: 10.3109/00365521.2011.551889, 21291294

[65] Ram M Barzilai O Shapira Y Anaya JM Tincani A Stojanovich L . *Helicobacter pylori* serology in autoimmune diseases - fact or fiction? Clin Chem Lab Med. (2013) 51:1075–82. doi: 10.1515/cclm-2012-0477, 23079514

[66] Sayar R Shokri Shirvani J Hajian-Tilaki K Vosough Z Ranaei M. The negative association between inflammatory bowel disease and *Helicobacter pylori* seropositivity. Caspian J Intern Med. (2019) 10:217–22. doi: 10.22088/cjim.10.2.217, 31363401 PMC6619465

[67] Chen Y Blaser MJ. *Helicobacter pylori* colonization is inversely associated with childhood asthma. J Infect Dis. (2008) 198:553–60. doi: 10.1086/590158, 18598192 PMC3902975

[68] Wjst M. Does *Helicobacter pylori* protect against asthma and allergy? Gut. (2008) 57:1178–9. doi: 10.1136/gut.2007.133462 18628381

[69] Arnold IC Hitzler I Müller A. The immunomodulatory properties of *Helicobacter pylori* confer protection against allergic and chronic inflammatory disorders. Front Cell Infect Microbiol. (2012) 2:10. doi: 10.3389/fcimb.2012.00010, 22919602 PMC3417532

[70] Bai X Jiang L Ruan G Liu T Yang H. *Helicobacter pylori* may participate in the development of inflammatory bowel disease by modulating the intestinal microbiota. Chin Med J. (2022) 135:634–8. doi: 10.1097/CM9.0000000000002008, 35234697 PMC9276318

[71] Ni J Wu GD Albenberg L Tomov VT. Gut microbiota and IBD: causation or correlation? Nat Rev Gastroenterol Hepatol. (2017) 14:573–84. doi: 10.1038/nrgastro.2017.88, 28743984 PMC5880536

[72] Abraham C Cho JH. Inflammatory bowel disease. N Engl J Med. (2009) 361:2066–78. doi: 10.1056/NEJMra0804647, 19923578 PMC3491806

[73] Podolsky DK. Inflammatory bowel disease. N Engl J Med. (2002) 347:417–29. doi: 10.1056/nejmra020831, 12167685

[74] Sakuraba A Sato T Kamada N Kitazume M Sugita A Hibi T. Th1/Th17 immune response is induced by mesenteric lymph node dendritic cells in Crohn's disease. Gastroenterology. (2009) 137:1736–45. doi: 10.1053/j.gastro.2009.07.049, 19632232

[75] Strober W Fuss IJ. Proinflammatory cytokines in the pathogenesis of inflammatory bowel diseases. Gastroenterology. (2011) 140:1756–67. doi: 10.1053/j.gastro.2011.02.016, 21530742 PMC3773507

[76] Wroblewski LE Peek RM Jr Wilson KT. *Helicobacter pylori* and gastric cancer: factors that modulate disease risk. Clin Microbiol Rev. (2010) 23:713–39. doi: 10.1128/CMR.00011-10, 20930071 PMC2952980

[77] Haskey N Gibson DL. An examination of diet for the maintenance of remission in inflammatory bowel disease. Nutrients. (2017) 9:259. doi: 10.3390/nu9030259, 28287412 PMC5372922

[78] Benchimol EI Manuel DG Mack DR Nguyen GC Gommerman JL. Asthma, type 1 and type 2 diabetes mellitus, and inflammatory bowel disease amongst south Asian immigrants to Canada and their children: a population-based cohort study. PLoS One. (2015) 10:e0123599. doi: 10.1371/journal.pone.0123599, 25849480 PMC4388348

[79] Franceschi C Campisi J. Chronic inflammation (inflammaging) and its potential contribution to age-associated diseases. J Gerontol A Biol Sci Med Sci. (2014) 69:S4–9. doi: 10.1093/gerona/glu057 24833586

[80] Malfertheiner P Megraud F Rokkas T Gisbert JP Liou JM Schulz C . Management of *Helicobacter pylori* infection: the Maastricht VI/Florence consensus report. Gut. (2022) 71:1724–62. doi: 10.1136/gutjnl-2022-327745 35944925

[81] Schwartzbaum J Ahlbom A Feychting M. Berkson's bias reviewed. Eur J Epidemiol. (2003) 18:1109–12. doi: 10.1023/b:ejep.0000006552.89605.c8 14758866

