# Supplementary material for: To eradicate or not? Helicobacter pylori in patients with inflammatory bowel disease: an updated systematic review and meta-analysis
Source: Front Med (Lausanne). 2026 Feb 3;13:1757356. doi: 10.3389/fmed.2026.1757356 (PMC12910842; doi:10.3389/fmed.2026.1757356)
Supplement: SUPPLEMENTARY TABLE 1 — Search Syntax of the Databases. [file Table_1.DOCX]

# Search Syntax of the Databases

## PubMed

((((((((((((((((((((("Crohn Disease"[Mesh]) OR (Crohn's Enteritis)) OR (Regional Enteritis)) OR (Crohn's Disease)) OR (Crohn's Disease)) OR (Inflammatory Bowel Disease 1)) OR (Enteritis, Granulomatous)) OR (Granulomatous Enteritis)) OR (Enteritis, Regional)) OR (Ileocolitis)) OR (Colitis, Granulomatous)) OR (Granulomatous Colitis)) OR (Ileitis, Terminal)) OR (Terminal Ileitis)) OR (Ileitis, Regional)) OR (Regional Ileitides)) OR (Regional Ileitis)) OR (("Colitis, Ulcerative"[Mesh]) OR (Idiopathic Proctocolitis) OR (Ulcerative Colitis) OR (Colitis Gravis) OR (Inflammatory Bowel Disease, Ulcerative Colitis Type))) OR ((("Inflammatory Bowel Diseases"[Mesh]) OR (Inflammatory Bowel Disease)) OR (Bowel Diseases, Inflammatory)))) AND ((((("Helicobacter pylori"[Mesh]) OR (Helicobacter nemestrinae)) OR (Campylobacter pylori)) OR (Campylobacter pylori subsp. pylori)) OR (Campylobacter pyloridis)))

## Embase

(('helicobacter pylori'/exp OR 'helicobacter pylori') OR ('campylobacter pylori subsp. pylori' OR 'campylobacter pyloridis' OR 'campylobacter pylori' OR 'helicobacter nemestrinae') OR ('campylobacter pylori' OR 'campylobacter pyloridis' OR 'campylobacter pyloris' OR 'helicobacter nemestrinae' OR 'helicobacter pylori')) AND ((('inflammatory bowel disease'/exp OR 'inflammatory bowel disease') OR ('inflammatory bowel disease' OR 'bowel diseases, inflammatory') OR ('inflammatory bowel diseases' OR 'inflammatory bowel disease')) OR (('crohn disease'/exp OR 'crohn disease') OR ('cleron disease' OR 'crohn`s disease' OR 'crohns disease' OR 'enteritis regionalis' OR 'intestinal tract, regional enteritis' OR 'morbus crohn' OR 'regional enteritis' OR 'regional enterocolitis' OR 'crohn disease') OR ('crohn`s enteritis' OR 'regional enteritis' OR 'crohn`s disease' OR 'crohns disease' OR 'inflammatory bowel disease 1' OR 'enteritis, granulomatous' OR 'granulomatous enteritis' OR 'enteritis, regional' OR 'ileocolitis' OR 'colitis, granulomatous' OR 'granulomatous colitis' OR 'ileitis, terminal' OR 'terminal ileitis' OR 'ileitis, regional' OR 'regional ileitides' OR 'regional ileitis')) OR (('ulcerative colitis'/exp OR 'ulcerative colitis') OR ('chronic ulcerative colitis' OR 'colitis ulcerativa' OR 'colitis ulcerosa' OR 'colitis ulcerosa chronica' OR 'colitis, mucosal' OR 'colitis, ulcerative' OR 'colitis, ulcerous' OR 'colon, chronic ulceration' OR 'histiocytic ulcerative colitis' OR 'mucosal colitis' OR 'ulcerative colorectitis' OR 'ulcerative procto colitis' OR 'ulcerative proctocolitis' OR 'ulcerous colitis' OR 'ulcerative colitis') OR ('colitis gravis' OR 'idiopathic proctocolitis' OR 'inflammatory bowel disease, ulcerative colitis type' OR 'ulcerative colitis' OR 'colitis, ulcerative')))

## Medline

(exp Inflammatory Bowel Diseases/ or (Inflammatory Bowel Disease or Bowel Diseases, Inflammatory).af.) or (exp Crohn Disease/ or (Crohn's Disease or Crohns Disease or Crohn's Enteritis or Inflammatory Bowel Disease 1 or Regional Enteritis or Ileocolitis or Ileitis, Terminal or Terminal Ileitis or Ileitis, Regional or Regional Ileitides or Regional Ileitis or Enteritis, Granulomatous or Granulomatous Enteritis or Enteritis, Regional or Colitis, Granulomatous or Granulomatous Colitis).af.) or (exp Colitis, Ulcerative/ or (Colitis Gravis or Idiopathic Proctocolitis or Inflammatory Bowel Disease, Ulcerative Colitis Type or Ulcerative Colitis).af.) and (exp Helicobacter pylori/ or (Campylobacter pylori subsp pylori or Campylobacter pyloridis or Campylobacter pylori or Helicobacter nemestrinae).af. )

## Web of Science

(Helicobacter pylori (Topic) OR Campylobacter pylori subsp. pylori (Topic) OR Campylobacter pyloridis (Topic) OR Campylobacter pylori (Topic) OR Helicobacter nemestrinae (Topic) and Preprint Citation Index (Exclude – Database)) AND ((Inflammatory Bowel Diseases (Topic) OR Inflammatory Bowel Disease (Topic) OR Bowel Diseases, Inflammatory (Topic) and Preprint Citation Index (Exclude – Database)) OR (Crohn Disease (Topic) OR Crohn's Disease (Topic) OR Crohns Disease (Topic) OR Crohn's Enteritis (Topic) OR Inflammatory Bowel Disease 1 (Topic) OR Regional Enteritis (Topic) OR Ileocolitis (Topic) OR Ileitis, Terminal (Topic) OR Terminal Ileitis (Topic) OR Ileitis, Regional (Topic) OR Regional Ileitides (Topic) OR Regional Ileitis (Topic) OR Enteritis, Granulomatous (Topic) OR Granulomatous Enteritis (Topic) OR Enteritis, Regional (Topic) OR Colitis, Granulomatous (Topic) OR Granulomatous Colitis (Topic) and Preprint Citation Index (Exclude – Database)) OR (Colitis, Ulcerative (Topic) OR Colitis Gravis (Topic) OR Idiopathic Proctocolitis (Topic) OR Inflammatory Bowel Disease, Ulcerative Colitis Type (Topic) OR Ulcerative Colitis (Topic) and Preprint Citation Index (Exclude – Database)))
