# Supplementary figures and images for: To eradicate or not? Helicobacter pylori in patients with inflammatory bowel disease: an updated systematic review and meta-analysis
Source: Front Med (Lausanne). 2026 Feb 3;13:1757356. doi: 10.3389/fmed.2026.1757356 (PMC12910842; doi:10.3389/fmed.2026.1757356)

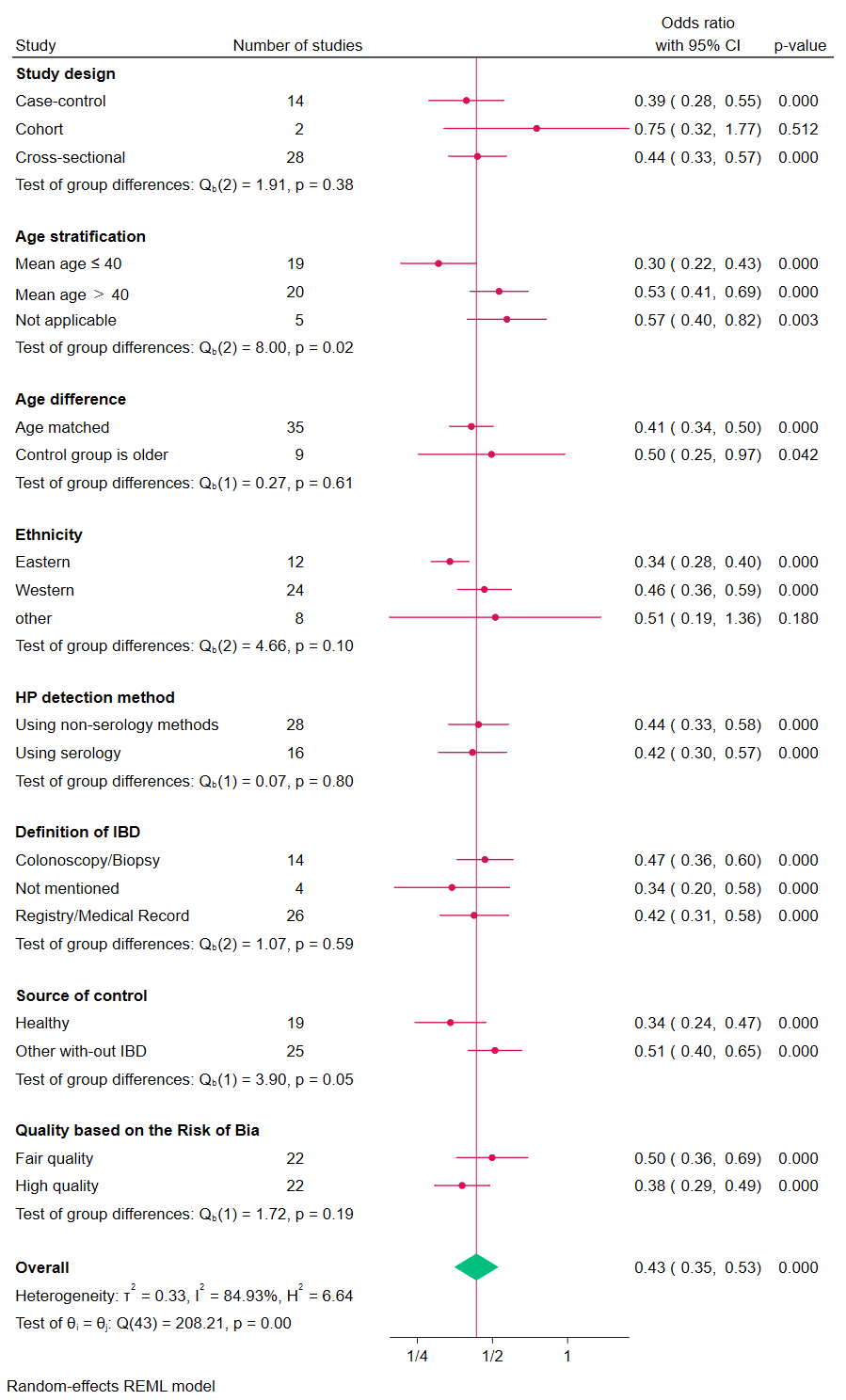

Supplement: SUPPLEMENTARY IMAGE 1 — Association between Hp infection and IBD stratified by subgroup. [file Image_1.tif]

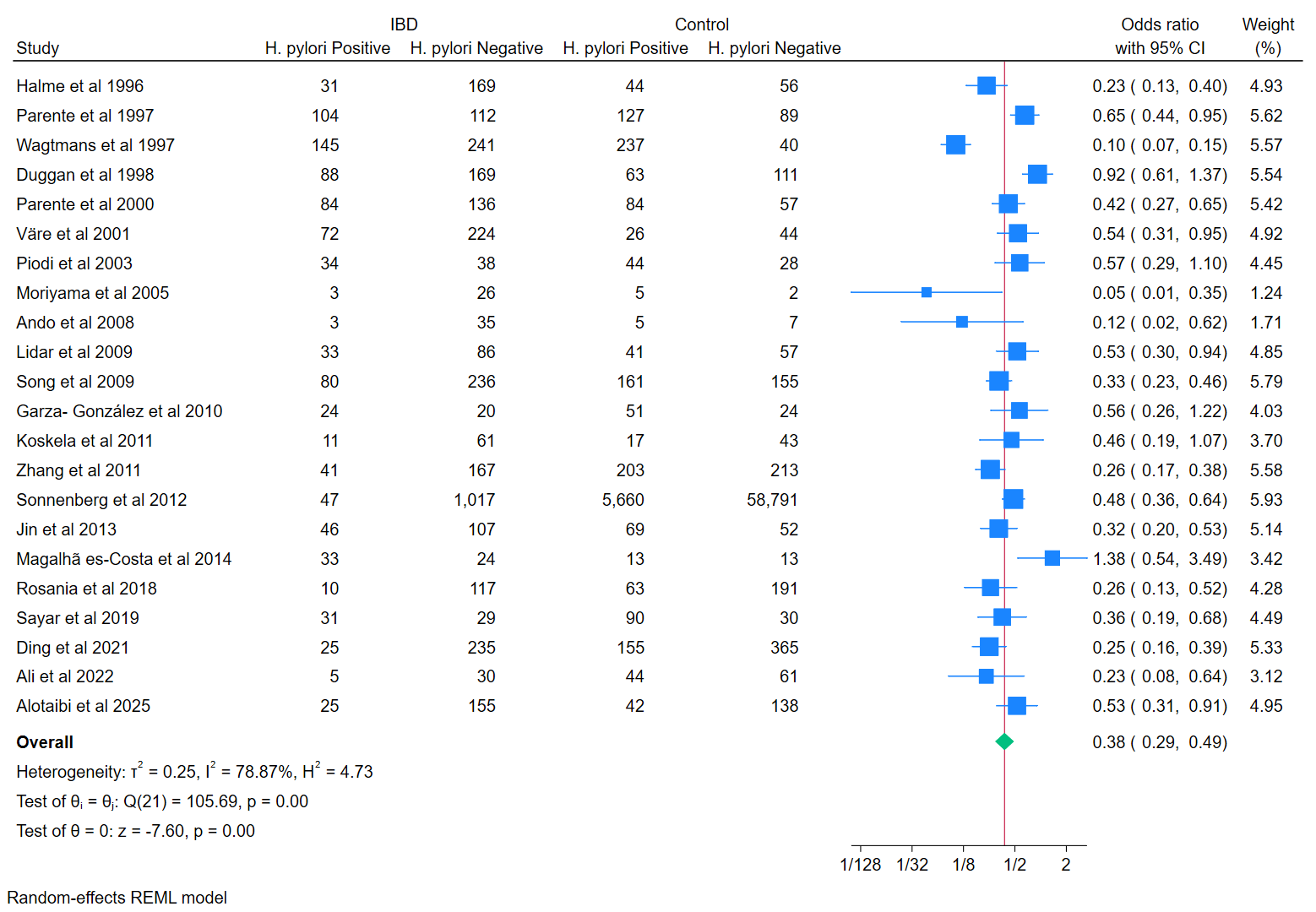

Supplement: SUPPLEMENTARY IMAGE 2 — Results of sensitivity analyses, excluded lower-quality studies. [file Image_2.TIF]

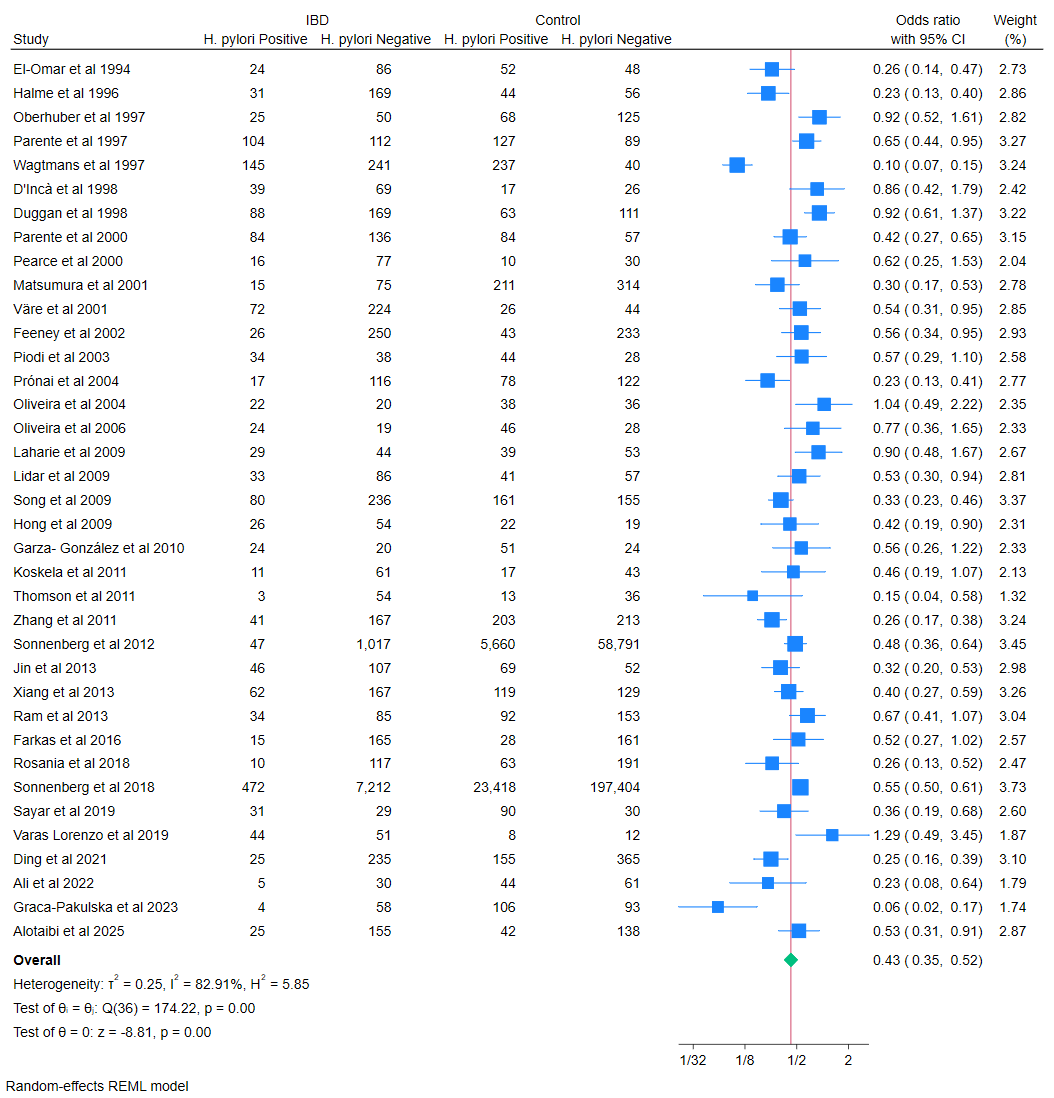

Supplement: SUPPLEMENTARY IMAGE 3 — Results of sensitivity analyses excluded studies with a sample size smaller than 100. [file Image_3.TIF]

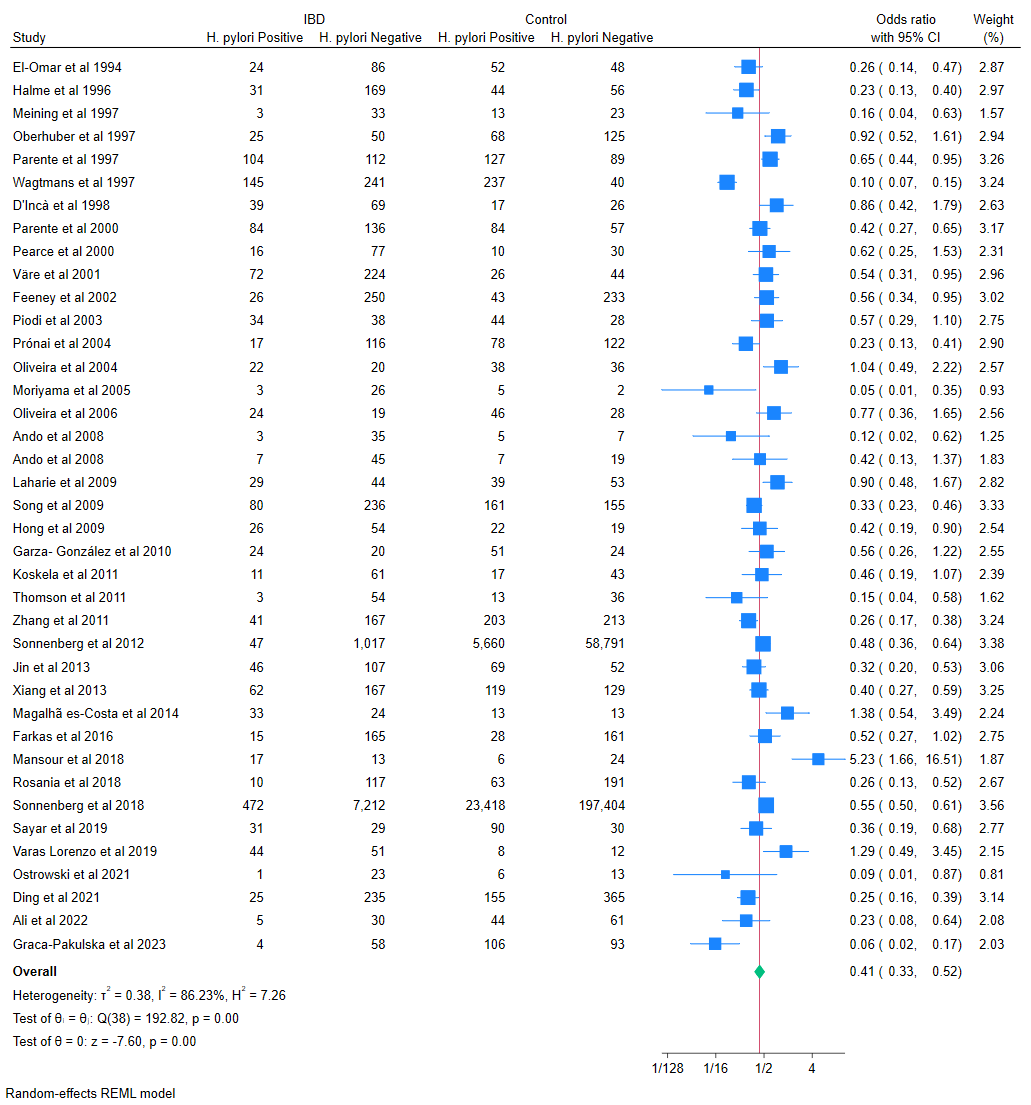

Supplement: SUPPLEMENTARY IMAGE 4 — Results of sensitivity analyses excluded studies with unclear age stratification. [file Image_4.TIF]
